# GAP between knowledge and skills for the implementation of the ACCM/PALS septic shock guidelines in India: Is the bridge too far?

**DOI:** 10.4103/0972-5229.56049

**Published:** 2009

**Authors:** Indumathy Santhanam, Niranjan Kissoon, S. R. Kamath, Suchitra Ranjit, Jayanthi Ramesh, Janani Shankar

**Affiliations:** **From:**1 Pediatric Emergency Department, Institute of Child Health and Hospital for Children, Madras Medical College, Chennai, India; 2 Acute and Critical Care Programs, Department of Pediatrics, University of British Columbia, Vancouver, British Columbia, Canada; 3 Pediatric Intensive Care Unit Mehta's Children's Hospital, Chennai; 4 Pediatric Intensive Care Unit, Apollo Hospitals, Chenna; 5,6 Kanchi Kamakoti Childs Trust Hospital, Chennai

**Keywords:** Septic shock, recognition, children, emergency department, ACCM guidelines, PALS, knowledge barriers, critical illness

## Abstract

**Objective:**

To determine whether physicians were aware of and had the skills to implement the American College of Critical Care Medicine/Pediatric Advanced Life Support Course septic shock protocol.

**Design:**

A cross-sectional questionnaire survey.

**Setting:**

Four academic institutions in Chennai, Manipal, Mangalore, and Trivandrum - cities representing the three southern states of Tamil Nadu, Karnataka, and Kerala, respectively, between February and April 2006.

**Interventions:**

Pre and post lecture questions. They were evaluated using 11 questions testing knowledge and 10 questions testing their comfort level in performing interventions related to the initial resuscitation in septic shock.

**Measurement and Main Result:**

The ACCM/PALS sepsis guidelines were taught during the PALS course conducted in the four academic institutions. A total of 118 delegates participated, of whom 114 (97%) were pediatricians and four (3%) were anaesthetists. The overall mean number of correct responses for the 11 questions testing knowledge before and after the lecture was 2.1 and 4.07, respectively *P*=0.001(paired t test). Although, 42% of the respondents (n=50) were aware of the ACCM guidelines, 88% (n=104) did not adhere to it in their practice. A total of 86% (n=101) and 66% (n=78) did not feel comfortable titrating inotropes or intubating in the ED; 78% (n=92) and 67% (n=78), respectively felt that central venous access (CVA) and arterial pressure (AP) monitoring were unimportant in the management of fluid refractory shock. Of the physicians, 20% (n=24) had never intubated a patient, 78% (n=92) had not introduced a central venous catheter, and 76% (n=90) had never introduced an intra-arterial catheter.

**Conclusions:**

In view of the lack of skills and suboptimal knowledge, the ACCM/PALS sepsis guidelines may be inappropriate in its current format in the Indian setting. More emphasis needs to be placed on educating community pediatricians with a simpler clinical protocol, which has the potential to save many more children.

## Background

The World Health Organization has reported that 60% of deaths in developing countries occurred as a result of communicable diseases.[1] A total of 50% of the deaths due to severe sepsis in these countries occurred within the first 24 hours of admission and often shock preceded death.[2] In India, a lack of responsive emergency medical systems,[3] late presentation with little pre-hospital resuscitation,[4] and very few well-equipped and appropriately staffed pediatric emergency departments (PED),[5] are some of the reasons contributing to the high mortality in pediatric septic shock. In addition, front-line physicians often fail to recognize early signs of septic shock resulting in the failure to institute appropriate therapy. Literature reports that when shock was unresolved, progression to multi-organ failure would be inevitable resulting in an overall mortality of 46% to 54%.[6–9]

Cognizant of the need for early recognition and treatment, a time sensitive, goal directed, step-wise protocol was published by the American College of Critical Care Medicine (ACCM)[10] to guide the bedside physician in the recognition and management of shock in the initial hours of presentation. This protocol was incorporated into the Pediatric Advanced Life Support (PALS) Course Manual in 2002.[11] Aggressive emergency management using this protocol has been successful in decreasing mortality from septic shock in various countries.[12–17] In 1993, the PALS was formally accredited by the Indian Academy of Pediatrics (IAP) as a separate cell of the IAP. Since then, this course has been conducted by certified PALS instructors.[18]

We chose to conduct this survey to determine whether physicians involved in the care of children were aware of the ACCM/PALS sepsis guidelines, and whether they had the skills necessary to implement the guidelines.

## Methods

The ACCM/PALS sepsis guidelines were taught as a separate interactive presentation during the PALS courses conducted in 4 academic institutions in Chennai, Manipal, Mangalore, and Trivandrum - cities representing the three southern states of Tamil Nadu, Karnataka, and Kerala, respectively, between February and April 2006. Various case scenarios were discussed in the shock work station. Each respondent was required to provide some demographic data and answer 11 clinical questions testing their knowledge and ten questions testing their comfort level in performing interventions related to the initial resuscitation in septic shock. The 7^th^ question had three sub-components testing the choice of inotrope in the different clinical scenarios in shock. We assigned a score of 1 for each correct answer resulting in a maximum score of 11. Acceptable answers were based on the recommendations provided by the ACCM/PALS sepsis guidelines 2002. A student t test was used to compare the mean scores of correctly answered individual questions pre and post lecture. A p-value of less than 0.05 was considered significant.

## Results

A total of 118 delegates participated of whom 114 (97%) were pediatricians and four (3%) were anesthetists. A total of 105 (89%) had less than three years of experience in their specialties. Eighty-two (75%) worked in teaching institutions that catered to both adults and children. Sixty-two (53%) physicians worked in hospitals with an outpatient census of less than 20,000, 35 physicians worked in hospitals that had between 20,000 to 30,000 patients in the out patient department (OPD), while 15 physicians (13%) were employed in institutions that register more than 40,000 patients every year. While all institutions treated children with serious sepsis, the number of children managed in each institution is unknown.

### (a) Knowledge

**Figure 1:**
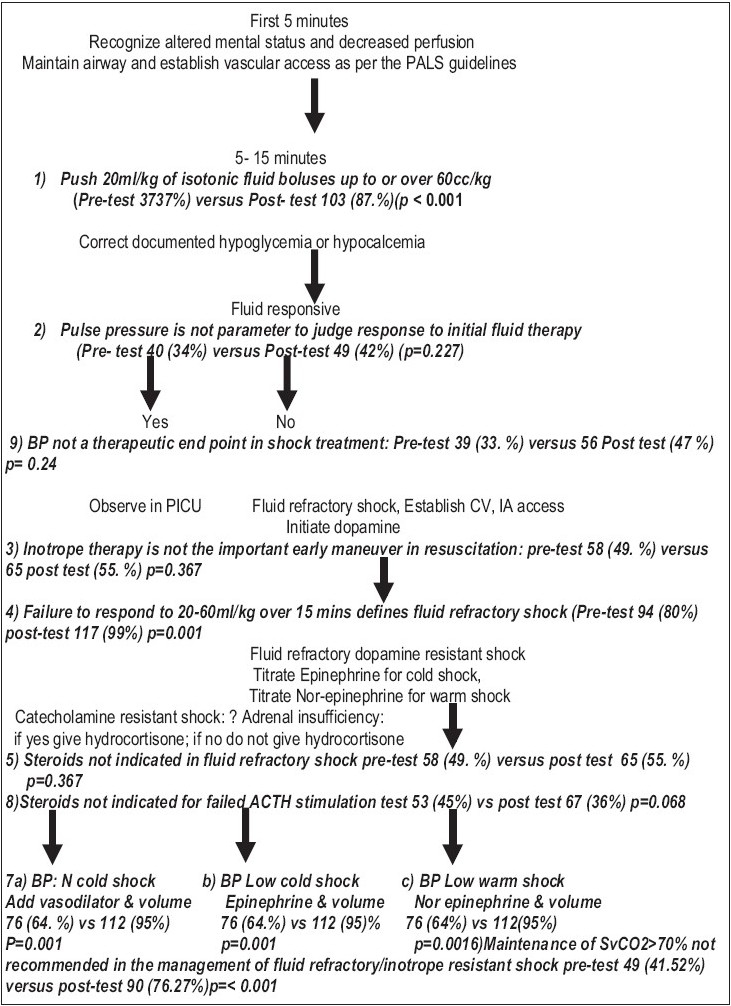
ACCM/PALS protocol for the management of septic shock in the ED

There were more correct answers to all questions post lecture than before the lecture [Table 1Figure 1]. The overall mean number of correct responses for the 9 questions testing knowledge before and after the lecture was 2.1 and 4.07, respectively (*P*=0.001 paired t test).

**Table 1:** Answers to questions posed in a pre- and post-questionnaire

Questions testing knowledge	Pre test	Post test	t	df	*P* value
					
	Mean ± SD	Mean ± SD			
1. Volume of fluids needed to correct shock in the first hour	0.05 ± 0.22	0.56 ± 0.50	10.32	118	0.000
2. Correct parameters used to judge response to the initial fluid therapy	0.56 ± 0.50	0.62 ± 0.49	1.15	118	0.252
3. Important early maneuvers in resuscitation of septic shock	0.26 ± 0.44	0.47 ± 0.50	3.92	118	0.000
4. Definition of fluid refractory shock	0.03 ±0.16	0.08 ±0.28	1.96	118	0.052
5. Interventions recommended for the management of fluid refractory shock	0.38 ±0.49	0.47 ±0.50	1.63	118	0.105
6. Management of dopamine/ dobutamine resistant septic shock	0.45 ±0.50	0.42± 0.49	0.54	118	0.588
7a. Choice of vaso dilator/ appropriate catecholamine in the management of fluid refractory dopamine unresponsive normotensive cold shock	0.02 ±0.13	0.08 ±0.27	2.14	118	0.034
7b. Choice of vaso dilator/ appropriate catecholamine in the management of fluid refractory dopamine unresponsive hypotensive cold shock	0.03 ± 0.16	0.08 ±0.28	2.14	118	0.034
7c. Choice of vaso dilator/ appropriate catecholamine in the management of fluid refractory dopamine unresponsive hypotensive warm shock	0.01 ± 0.09	0.08 ± 0.28	3.11	118	0.002
8. The indication of corticosteroids in the management of septic shock	0.26 ± 0.44	0.56 ± 0.50	5.47	118	0.000
9. Appropriate therapeutic endpoints for shock resolution	0.52 ± 0.50	0.65 ± 0.48	2.66	118	0.009
Total	2.51 ± 1.66	4.07 ±2.07	−8.50	118	0.000

### (b) Skills and Attitude

Although 42% respondents (n=50) were aware of the ACCM/PALS sepsis guidelines, 88% (n=104) did not adhere to it in their practice. However, 98% (n=115) were interested in learning the protocol. A total of 86% (n=101) and 66% (n=78), respectively did not feel comfortable titrating inotropes or intubating in the ED, 78% (n=92) and 67% (n=78), respectively felt that CVA and AP monitoring were unimportant in the management of fluid refractory shock. Of the physicians, 20% (n=24) had never intubated a patient, 78% (n=92) had not introduced a CV catheter, and 76% (n=90) had never introduced an arterial pressure catheter [Table 2].

**Table 2:** Comfort levels and confidence in performing the following maneuvers in the ED

Competence and perceptions	Yes		No	
		
	No	%	No	%
Performed central venous cannulation	76	64.4	43	35.6
Performed intra-arterial cannulation	87	73.7	31	26.3
Initiation of inotropes	17	14.4	101	85.6
Titration of inotropes	36	30.5	82	69.5
Intubation	40	33.9	78	66.1
Need for CVP monitoring	26	22.0	92	78.0
Need IAP monitoring	39	33.1	79	66.9
Aware of pediatric sepsis protocol	50	42.4	68	57.6
Use of pediatric sepsis protocol	14	11.9	104	88.1
Need to learn more about the treatment of sepsis	3	2.5	115	97.5

## Discussion

We conducted this survey to determine the knowledge of the ACCM/PALS sepsis guidelines, the skills needed to treat sepsis using these guidelines, and the immediate impact of an interactive presentation. Although, we found modest improvement in knowledge following the lecture, physicians lacked the skills needed to implement this protocol.

On recognition of septic shock, the ACCM/PALS 2002 sepsis protocol recommended attention to the airway, establishment of ventilation, and administration of 20 ml/kg fluid boluses up to or more than 60 ml/kg. If shock was still refractory, it suggested the initiation of an inotrope and placement of CVA and AP catheters to estimate ventricular filling pressures.[19]

Various barriers unique to India prevent the successful translation of these guidelines to practice. Although the ACCM/PALS septic shock guidelines were published in Indian text books,[20–22] contemporary resuscitation in the PED is not widely known. Typically, emergency care in India is provided in areas known as “casualty” where infra-structure is poor.[23,24] Specialty training is neither available nor mandatory for personnel involved in Pediatric Emergency Medicine (PEM). Besides, currently there is no Medical Council of India accredited residency program in emergency medicine.[3] Although there are a few hospitals with organized PED services, these are located in large cities affiliated with corporate hospitals and a few apex medical schools. Mostly, the majority of institutions catering to the economically disadvantaged children in India do not have separate PEDs.[8] Furthermore, the medical curriculum fails to place sufficient emphasis on resuscitation training in PEM.[25]

The lack of hands-on experience and comfort levels in performing procedures such as intubation, CVA or AP catheter placement, and resuscitation pharmacology is therefore not surprising. Skippen, *et al*. observed that the ACCM/PALS sepsis guidelines cannot be used in environments that lack the skills necessary to implement the guidelines.[26] Our findings lend evidence to this observation. In fact, although, these guidelines were nicely laid out, even community pediatricians in the USA were found lacking in their performance of critical care procedures and in the coordination of the temporal aspects of a prolonged resuscitation.[19]

Predictably therefore, the death rates in severe sepsis cases reported by apex pediatric intensive care units in India are high at 30 to 50%.[27–29] The surviving sepsis campaign for 2008 envisages a global fall in mortality to 10%.[30] In order to move closer to this goal, a protocol less dependent on invasive monitoring and more dependent on clinical assessment may be more appropriate in the Indian context.[4] Indeed, class 1a evidence from an academic PED of a government children's hospital in Southern India showed that administration of fluids at the rate of 20 ml/kg over 20 minutes up to 60 ml/kg of fluid in the first hour and initiation of an inotrope and performance of intubation when “intubation triggers” were identified followed by further fluids boluses until clinical goals of shock resolution were achieved had demonstrated a dramatic drop in mortality from 50% to 17.6% (*P*=0.0001) 95%; CI 11.9-24.8%. This data also established the impact of meticulous and serial clinical cardiopulmonary assessment on survival. (OR for survival if shock due to sepsis was corrected in the initial hour was 9.2; 95% CI, 2.1-40.8).[4]

Another major barrier highlighted by our survey was our inability to provide a reasonable educational experience even for those who chose to seek this knowledge. The experience from other continuing medical education strategies has also not been encouraging. A review of 32 studies, with almost 3,000 health professionals[31] found didactic teaching to be ineffective, interactive workshops to be moderately effective and combining workshops with didactic learning only moderately effective. In a Canadian report of 17 studies examining all resuscitation courses, five showed no improvement in knowledge and eight showed no improvement in skills retention.[32] In a study from Baltimore, 45 PALS trained doctors showed poor performance for skills and prolonged time to skill completion.[33] In another study, successful performance improved for airway management, intra-osseous access, and defibrillation immediately after completion of the PALS course.[34] Pediatric residents were confident in their ability to manage emergencies in two surveys.[35,36] However, when they were examined, none of the residents were able to successfully perform both basic and advanced airway skills, and only 11% completed two vascular skills. In the UK, a survey of 88 pediatricians reported poor knowledge in resuscitation with only 9% having had training in PEM.[35] In a later survey, only 26% of 57 physicians (1/3^rd^ who had training in PEM) provided satisfactory answers to questions about cardiac arrest protocols.[36] These surveys, however, did not include the PALS/ACCM sepsis guidelines and cannot be directly compared with our current study. Computerized mannequins with realistic cardio-respiratory responses may improve mastering of skills in emergency care.[37,38] The expense and non availability of these mannequins limit its use in the predominantly non-profit PALS courses conducted in India.

However, despite the barriers to knowledge transfer, the eagerness of virtually all delegates to learn current concepts is heartening. Perhaps, inclusion of a module demonstrating the management of septic shock using clinical goals[4] may be more appropriate in the Indian context.[39]

## Conclusion

In view of the lack of skills and suboptimal knowledge, the ACCM/PALS sepsis guidelines may be inappropriate in its current format in the Indian setting. More emphasis needs to be placed on educating community pediatricians with a simpler clinical protocol that has the potential to save many more children.
